# Serotonergic modulation of normal and abnormal brain dynamics: The genetic influence of the *TPH*2 *G-703T* genotype and DNA methylation on wavelet variance in children and adolescents with and without ADHD

**DOI:** 10.1371/journal.pone.0282813

**Published:** 2023-04-27

**Authors:** Atae Akhrif, Marcel Romanos, Katharina Peters, Ann-Kathrin Furtmann, Julian Caspers, Klaus-Peter Lesch, Eva M. Meisenzahl-Lechner, Susanne Neufang

**Affiliations:** 1 Department of Psychiatry and Psychotherapy, Medical Faculty, Heinrich-Heine-University Duesseldorf, Duesseldorf, Germany; 2 Department of Child and Adolescent Psychiatry, Center of Mental Health, University Hospital Wuerzburg, Wuerzburg, Germany; 3 Department of Diagnostic and Interventional Radiology, Medical Faculty, Heinrich-Heine-University Duesseldorf, Duesseldorf, Germany; 4 Division of Molecular Psychiatry, Center of Mental Health, University of Wuerzburg, Wuerzburg, Germany; 5 Department of Psychiatry and Neuropsychology, School for Mental Health and Neuroscience, Maastricht University, Maastricht, The Netherlands; UCSI: UCSI University, MALAYSIA

## Abstract

Attention-deficit/hyperactivity disorder (ADHD) is a neurodevelopmental disorder that often persists into adulthood. Core symptoms of ADHD, such as impulsivity, are caused by an interaction of genetic and environmental factors. Epigenetic modifications of DNA, such as DNA methylation, are thought to mediate the interplay of these factors. Tryptophan hydroxylase 2 (TPH2) is the rate-limiting enzyme in brain serotonin synthesis. The TPH2 gene has frequently been investigated in relation to ADHD, e.g., showing that *TPH2 G-703T* (rs4570625) polymorphism influences response control and prefrontal signaling in ADHD patients. In this (epi)genetic imaging study we examined 144 children and adolescents (74 patients, 14 females) using fMRI at rest and during performing a waiting impulsivity (WI) paradigm. Both, *TPH2 G-703T* (rs4570625) genotype and DNA methylation in the 5’ untranslated region (5’UTR) of *TPH2* were associated with wavelet variance in fronto-parietal regions and behavioral performance, taking *TPH2* genotype into account. In detail, comparisons between genotypes of patients and controls revealed highest wavelet variance and longest reaction times in patients carrying the T allele [indicative for a gene-dosage effect, i.e., the WI phenotype is a direct result of the cumulative effect of ADHD and *TPH2* variation]. Regressions revealed a significant effect on one specific DNA methylation site in ADHD patients but not controls, in terms of a significant prediction of wavelet variance in fronto-parietal regions as well as premature responses. By the example of the *TPH2 G-703T* (rs4570625) polymorphism, we provide insight into how interactive genetic and DNA methylation affect the ADHD and/or impulsive endophenotype.

## Introduction

Attention-Deficit/Hyperactivity Disorder (ADHD) is the most prevalent neurodevelopmental disorder [1], characterized by inattention, hyperactivity, and increased impulsivity [2]. Besides the neurotransmitters dopamine and noradrenaline, serotoninergic neurotransmission seems to play an important role in ADHD as genetic association studies identified serotonergic system gene variants to be associated with ADHD phenotype [3, 4]. For example, in patients with ADHD impulsive behavior [5] as well as neural functioning at rest and under task varied between genotypes of serotonergic genes [6]. Additionally, pharmacological studies proved the efficiency of serotonin-noradrenaline reuptake inhibitors in the treatment of ADHD [7]. However, the role of serotonin has often been discussed as indirect, e.g. via the interaction with dopamine [3, 8], its higher impact in ADHD patients with comorbid depression [9], or in terms of gene-by-environment / gene-by-brain interaction. For example, van der Meer and colleagues (2017) compared functional connectivity of the resting-state networks fronto-parietal executive control and default mode network (FPN, DMN) between ADHD patients reporting an (S-)allele-specific manifestation of stress exposure in terms of a decrease in FPN connectivity/cognitive control and an increase in DMN connectivity /rumination with increasing stress [6]. In an earlier study, we found that the influence of serotonin-synthesizing tryptophan-hydroxylase-2 (TPH2) *TPH2 G-703T* polymorphism on the aggressive phenotype was mediated by structure and function of the right prefrontal cortex (PFC) [10].

Gene expression is, furthermore, defined by *TPH2*’s DNA methylation levels [11, 12]. DNA methylation is an epigenetic modification in which a methyl group is added to cytosine in cytosine–phosphate–guanine (CpG) sites. This process can directly affect the activity and function of a gene without altering the DNA sequence, i.e., methyl-binding proteins may repress gene expression. For the *TPH2* gene, it was reported that methylation of a single CpG site in the promoter region of *TPH2* moderates gene expression levels [9], with low methylation resulting in high TPH2 activity and subsequently in high serotonin levels. First methylation analyses revealed, that lower DNA methylation levels in serotonergic genes were associated with more ADHD symptoms [13, 14] and a first epigenome-wide association study (EWAS) with ADHD patients did not find any significant associations between ADHD subtypes, and/or impulsive traits and CpG sites, which, however, might have been due to the small sample size [15].

However, one hallmark in the characterization of pathological processing in ADHD is that attention skills and/or impulsive behavior are not impaired *per se* but more inconsistent and with higher variability compared to typically developing children (TDC). Increased variability in ADHD patients has been found in reaction times [16], as well as resting-state fMRI (rs-fMRI) brain signals as measured e.g., via multiscale entropy [17] or the so called mean-square successive difference, a measure of moment-to-moment brain signal variability [18]. High variability has been assumed to reflect occasional lapses in attention, linked to intrusions of distracting activity during task performance and/or reduced anti-correlation between regions in the DMN and attention networks, as summarized in the *default mode interference hypothesis* [19]. Empirical evidence of the latter, however, was inconclusive as hypo-connectivity as well as hyper-connectivity were reported [20, 21], a combination of both [22] and any difference at all [23]. Therefore, Dajani et al. (2019) concluded that it is more likely the dynamics between and within neural networks [i.e., the variability of network processing across time- and frequency-scales], that are affected in ADHD, than functional connectivity [in terms of one coefficient describing the (averaged) correlation between two regions over time]. Therefore, recent studies introduced new approaches to quantify these dynamics such as dynamic functional connectivity using temporal and frequency-specific information of fMRI timeseries [24].

FMRI timeseries are the intensity values of the BOLD signal of a certain voxel/region of interest (ROI) at each data acquisition time point of the fMRI sequence. Thus, the graphical representation of fMRI timeseries shows the changes of the BOLD signal over the course of the scanning time. The BOLD signal, in return, reflects the underlying activity of neurons [25], with its amplitudes indicating increases in neural activity. As brain function is characterized by continuous cognitive processing, the BOLD signal of a certain ROI is the result of one or multiple, simultaneously running processes. Simultaneous processing is possible e.g., by using different frequency bands. For example, the conventional fMRI data acquisition bandwidth covers frequencies from 0 to 0.25Hz. Functional components of the BOLD signal have been divided into rs-fMRI-related low-frequency fluctuations [< .08Hz, 26] and cognitive-related frequencies e.g., in attention networks [>0.8Hz, [27]]. In an earlier study we found that signals emerging from prefrontal areas and going to parietal regions were at cognitive-related frequencies (0.08–0.15Hz), whereas signals coming from parietal regions and going to prefrontal areas, were associated with low frequencies (0.001–0.03Hz) [28]. Therefore, to isolate simultaneous processes based on their frequencies, the wavelet transform method is ideal for performing frequency band- or scale-based decomposition analyses [2.5 Eq (c)]. Wavelet variance (wVar) determines the variance of the BOLD signal scale-specifically using the temporal and frequency-specific information of fMRI timeseries [29, 30]. So, tracking the scale-based variance of the amplitude detects not only changes in the intensity of an activity as in comparing rs-fMRI versus task-fMRI, but also reveals the process-specific timescale(s) of simultaneous neural processes, i.e. brain dynamics [31]. Up till now, the dynamics of intrinsic neural networks have predominantly been studied at rest. Only one study addressed within-network variance in the DMN under task processing reporting that it was significantly higher in patients with ADHD. In a second step, DMN network variance was related to task performance revealing that low DMN variance was associated with high accuracy. The authors concluded that patients were less able to sustain DMN suppression during performing the task [32]. Serotonergic modulation of dynamic processes has not been studies, to date.

To summarize, the dynamic interplay between network regions is crucial in the pathology of ADHD. In this paper, we introduce a novel marker, wVar, to quantify these dynamics. We determined wVar at rest and under task in fMRI timeseries of the DMN and the FPN in three different frequency bands: 0.02 to 0.04Hz, 0.04 to 0.08Hz and 0.08–0.16Hz. The paradigm was the 4-choice serial reaction time task (4-CSRTT) [33], measuring waiting impulsivity (WI), which combines sustained attention and action restraint and is defined as the ability to inhibit a premature response in order to earn a reward. In a first step, we compared wVar coefficients between *fMRI conditions* (task vs. rest) and *groups* (ADHD vs. TDC). Assuming highly varying neural signaling at rest and low wVar during focused cognitive processing, we expected to find

In TDC

- in the cognitive frequencies of the FPN high wVar at rest and a decrease in wVar during task.
- in the low frequencies 0.02–0.04Hz in the DMN a reverse pattern (rest > task).
- In ADHD
- high wVar in the DMN during task processing based on the impaired DMN suppression in ADHD [34]
- high wVar in the FPN during task processing based on general higher variability in cognitive processing [16]

The relation between wVar and cognitive load was addressed to prove that wVar reflected even immediate changes in cognitive processing. We expected to find an increase in wVar with cognitive load predominantly in high frequencies of FPN regions accompanied by a potential decrease in task performance across both groups [32]. Finally, correlations between wVar and behavioral parameters were performed to directly link both parameters assuming significant correlations between wVar and accuracy with the lower wVar the higher accuracy [32].

After proving the sensitivity of wVar towards functional alterations in ADHD, we examined the effect of both, *TPH2 G-703T* genotype and 5’ untranslated region (5’UTR) methylation of *TPH2* on WI processing. Even though both, genetic variants and DNA methylation are being studied in an (epi)genome-wide approach nowadays [15, 35], and large genome-wide association studies have revealed only modest effects of single genetic variation for ADHD [36], we use the candidate gene approach in this study to dedicate all analyses to the mechanism of how (epi)genetic variation effects brain/behavior relation, i.e. to link (epi)genetic *TPH2 G-703T* variation to the ADHD/WI phenotype in a fine-grained manner.

For genotype analyses 2x2 MANOVA models were defined. Based on the finding, the TPH2 expression is decreased in carriers of the T allele [37], we expected to find a *gene-dosage effect* (GG_TDC_ vs. T^+^_TDC_ vs. GG_ADHD_ vs. T^+^_ADHD_), i.e. the WI phenotype is a direct result of the cumulative effect of WI phenotype (ADHD>TDC) and genetically moderated *TPH2* variation (T^+^>GG). Finally, the effect of DNA methylation on WI was examined as suggested by Reuter et al. (2020) [38] using multiple regressions taking *TPH2* genotype as well as age and sex into account. We predicted a positive relation between DNA methylation and wVar in terms of the higher methylation, the higher wVar. Whether genetic effects were frequency-specific, i.e., predominantly in cognitive and/or rs-fMRI associated frequencies, was an exploratory question.

## Materials and methods

### Participants

In total, 144 children and adolescents with and without ADHD were examined (14 females), comprising 70 patients with ADHD (8 females) and 74 typically developing children (TDC, 6 females). Six ADHD patients were excluded from statistical analysis as two patients did not finish MRI scanning and four patients presented extensive motion artifacts. Remaining 138 subjects were aged from 8 to 18 years. M = 12.8±2.3yrs, intelligence was screened via the "Culture Fair Intelligence Test" (M = 105.0±15.0, range: 80–153) (for sample-specific information see S1 Table). Healthy participants were recruited within the Collaborative Research Center TRR-58, and patients with ADHD were recruited from in/outpatient clinics of the Departments of Child and Adolescent Psychiatry at the University Hospital Wuerzburg and the LVR Hospital Duesseldorf. All patients were diagnosed with ADHD according to the DSM-V by trained clinicians (disease duration: M = 4.2±2.6yrs). Thirty-six patients were medicated with methylphenidate (20 long-acting, daily dosage: M = 32±11.1mg; medication duration: M = 2.3±2.5yrs), three with Lisdexamphetamin (daily dosage: M = 50mg), and one with atomoxetine (daily dosage = 18mg). Unmedicated patients were either medication naïve (n = 10) or stopped medication use for more than a year (n = 14). Seventeen patients were diagnosed with comorbid oppositional defiant disorder (F91.3), seven patients with additional dyslexia (F81.0), three with comorbid obsessive-compulsive disorder and one with Asperger syndrome. Affective comorbidities were diagnosed in nine patients [social phobia (F40.1): N = 1, childhood emotional disorder with social anxiety (F93.2): N = 1, childhood emotional disorder with sibling rivalry (F93.3): N = 1, other childhood emotional disorder (F93.8): N = 3, depression (F32.1): N = 3].

Medicated patients underwent a washout phase of min 48hrs prior to scanning. The study was in accordance with the Declaration of Helsinki in its latest version and was approved by the ethics committees of the Faculty of Medicine, University of Wuerzburg (No 238–14), and of the Medical Faculty, Heinrich-Heine-University Duesseldorf, Germany (No 2018–306). Participants and their parents/legal guardians gave written informed consent.

### Genotyping and assessment of DNA methylation

Genomic DNA was extracted from whole-blood samples according to a standard desalting protocol. Genotyping procedures were performed using PCR and gel electrophoresis. Genotyping for the *TPH2 G-703T* (rs4570625) polymorphism was performed according to published protocols [39]. TPH2 G-703T distribution (TT = 4.5%; GT = 50%; GG = 48.5%; p(Exact) = .7348) did not deviate significantly from the expected numbers calculated according to the Hardy–Weinberg equilibrium using the program DeFinetti provided (https://wpcalc.com/en/equilibrium-hardy-weinberg/). Based on the findings showing that TPH2 expression is decreased in carriers of the T allele [37], we defined two groups, subjects homozygous for the TPH2 G allele (GG) and carriers of at least one T allele (T^+^).

**Fig 1:**
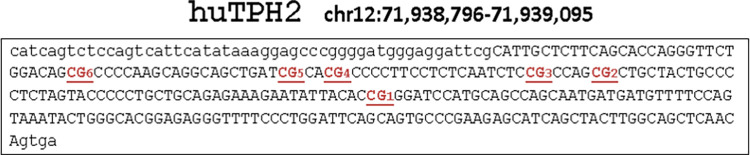
Sequence overview. Fig 1 represents the location of CpG sites, of which methylation was determined.

For DNA methylation assessment, aliquots of genomic DNA (250ng) were treated with sodium bisulfite by means of the EZ-96 DNA Methylation Kit (ZymoResearch, Freiburg, Germany). The Infinium MethylationEPIC Kit was used to quantify DNA methylation at ~865,000 sites (Illumina, San Diego, USA). Hybridization and processing were performed according to the manufacturer’s instructions (for promoters, starter sequence and location of CpG sites, see Fig 1).

### Paradigm

The used paradigm was an adapted version of the 4-CSRTT and has been described in detail in an earlier publication [10]. In the task, subjects were instructed to indicate the position of a briefly presented visual target after a waiting period and to respond as fast and as correct as possible to earn a reward. The task consisted of 5 blocks of increasing task difficulty:

block 1: target presentation duration was 64ms, cue target interval was 2000ms across all trials.

block 2 was so called baseline block, without rewarding and for motivation index/fatigue measurement purposes only (motivation index = reaction times [baseline block2]–reaction times [baseline block outside the scanner])

block 3: reduction of the target’s presentation duration from 64ms to 32ms;

block 4: short target presentation + variation of the cue-target interval between 2000ms and 6500ms; block 5: short target presentation + variation of the cue-target interval + inclusion of distractor targets.

Based on the block design and hierarchical structure of the 4-CSRTT, we were able to determine wVar and behavioral performance for each block separately (for details see S1 Fig).

### Data acquisition

Scanning was performed on a 3 Tesla TIM Trio Scanner at the Institute for Diagnostical and Interventional Neuroradiology at the University Hospital Wuerzburg and on a 3 Tesla PRISMA Scanner (Siemens, Erlangen, Germany) at the Department of Diagnostic and Interventional Radiology at the University Hospital Duesseldorf. Whole-brain T2*-weighted BOLD images were recorded with a simultaneous multi-slice echo-planar imaging sequence (repetition time = 800ms, echo time = 37ms, 72 slices, 2mm thickness, flip angle = 52°, rs-fMRI: 6:58min, 512volumes, task-fMRI: 14.5min, 1069 volumes).

### Data processing

Data processing was performed using the Functional Connectivity Toolbox (https://www.nitrc.org/projects/conn) as implemented in the Statistical Parametric Mapping Software Package (SPM12, http://www.fil.ion.ucl.ac.uk/spm/). Data preprocessing included temporal and spatial alignment (slice time correction, realignment and unwarping), spatial normalization into a standard stereotaxic space (MNI TPM template), resampling to an isotropic voxel size of 2×2×2mm^3^, outlier detection via ART-based scrubbing and spatial smoothing with a Gaussian kernel of 8mm full width at half maximum. The extraction of averaged timeseries was performed for regions of the DMN and FPN defined from CONN’s ICA analyses of the HCP dataset (497 subjects):

- DMN [medial prefrontal cortex, DMN.MPFC (1,55,-3), bi-hemispheric lateral parietal cortex, DMN.LP (-39,-77,33), (47,-67,29), and posterior cingulate cortex DMN.PCC (1,-61,38)]
- FPN [lateral prefrontal cortex, FPN.LPFC (-43,33,28), (41,38,30) and posterior parietal cortex FPN.PPC (-46,-58,49) (52,-52,45)].

Timeseries were corrected for movement artifacts using realignment parameters as covariates and extracted for each subject separately.

### wVar

In this section we briefly introduce wavelet transform from a signal filtering perspective leading to the definition of the wVar quantity introduced by Percival (1995) [29], which is based on wavelet transform coefficients. For detailed study and deeper theoretical insights of this topic and its use in praxis please refer to [40].

Given a timeseries $X\equiv \{X_{t}:t=0,\ldots ,N-1\}$ of length *N* and a set of mutually corresponding high and low frequency filters (sometimes called split-band filters) ${\tilde{h}}_{l}$ and ${\tilde{g}}_{l}$ of even length *L*, the circular linear filtering of *X* with $\left\{{\tilde{h}}_{\left(j,l\right)}:l=0,\ldots ,L_{j}-1\right\}$ and $\left\{{\tilde{g}}_{\left(j,l\right)}:l=0,\ldots ,L_{j}-1\right\}$ for *j* = 1,…,*J*_0_ where, *J*_0_<*log*_2_(*N*) and *L*_*j*_ ≡ (2^*j*−1^)(*L*−1)+1, produces *J*_0_ series of wavelet coefficients of length *N* each [Eq (1)]

$$\left\{ \begin{array}{c} W_{j,t}=\sum_{l=0}^{L_{j}-1}{\tilde{h}}_{j,l}X_{t-lmodN},t=0,\ldots ,N-1\\ {\tilde{h}}_{j,l}=h_{j,l}/2^{j/2}j=1,\ldots ,J_{0}\\ h_{j,l}:h_{j,0},h_{j,1},\ldots ,h_{j,L_{j}-2},h_{j,L_{j}-1}=h_{0},0,\ldots ,0,h_{1},0,\ldots ,0,h_{L-2},0,\ldots ,0,h_{L-1}\\ with\;(2^{j-1}-1)\;zeros\;between\;consecutive\;elements \end{array}\right.$$ (1)

and a single set of scaling coefficients of length *N* [Eq (2)]

$$\left\{ \begin{array}{c} V_{J_{0},t}=\sum_{l=0}^{L_{J_{0}}-1}{\tilde{g}}_{J_{0},l}X_{t-lmodN},t=0,\ldots ,N-1\\ {\tilde{g}}_{J_{0},l}=g_{J_{0},l}/2^{J_{0}/2}\\ g_{J_{0},l}:g_{J_{0},0},g_{J_{0},1},\ldots ,g_{J_{0},L_{J_{0},}-2},g_{J_{0},L_{J_{0},}-1}=g_{0},0,\ldots ,0,g,0,\ldots ,0,g_{L-2},0,\ldots ,0,g_{L-1}\\ with\;{(2}^{\left(J_{0}-1\right)}-1)\;zeros\;between\;consecutive\;elements \end{array}\right.$$ (2)

${\tilde{h}}_{j,l}$ and ${\tilde{g}}_{j,l}$ are called the *j*^*th*^ level maximal overlap discrete wavelet transform (MODWT), wavelet and scaling filter respectively.

The new *J*_0_+1 timeseries represent the *MODWT* of *X* up to level *J*_0_ if the applied filters fulfill the following criteria: given the notations *h*_1,*l*_ ≡ *h*_*l*_ and *g*_1,*l*_ ≡ *g*_*l*_ we have

$$\left\{ \begin{array}{c} \sum_{l=0}^{L-1}h_{l}=0,\sum_{l=0}^{L-1}h_{l}^{2}=1\\ \sum_{l=0}^{L-1}h_{l}h_{l+2n}=\sum_{l=-\infty }^{\infty }h_{l}h_{l+2n}=0 \end{array}\right.$$ (3)

as well as

$$\left\{ \begin{array}{c} \sum_{l=0}^{L-1}g_{l}=\sqrt{2},\sum_{l=0}^{L-1}g_{l}^{2}=1\\ \sum_{l=0}^{L-1}g_{l}g_{l+2n}=\sum_{l=-\infty }^{\infty }g_{l}g_{l+2n}=0 \end{array}\right.$$ (4)

for all nonzero integers *n*. That is, the filter coefficients must sum to zero, filter have the unit energy and even shifts implementations are orthogonal.

{*g*_*l*_} is the quadrature mirror filter that corresponds to {*h*_*l*_} that is

$g_{l}\equiv {\left(-1\right)}^{l+1}h_{L-1-l}$ and the inverse relationship is given by $h_{l}={\left(-1\right)}^{l}g_{L-1-l}$.

Let $U(f)=|U(f)|e^{\theta (f)}$ be the transfer function of a filter, *f* the frequency and *θ*(*f*) the phase function. Because the before mentioned conditions do not necessarily result in a unique set of filters, although they are only different in their phase functions, additional constraints are added to define a sub-class of filters making the interpretation of the resulting transform coefficients and the associated j level filters meaningful:

- level *j* wavelet coefficients {*W*_*j*_} are differences between weighted averages of *X* values localized within scales (time windows) of length *τ*_*j*_ ≡ 2^*j*−1^*Δt* (with *Δt* = 0.8*sec*, the repetition time of fMRI sequence) and
- level *j* wavelet filters {*h*_*j*_} are good approximation to band-pass filters and therefore the filter output coefficients are related to frequency bands $[1/2^{j+1},1/2^{j}]\frac{1}{\Delta t}$ (see S2 Fig).
- the vector $V_{J_{0}}$ contains the scaling coefficients associated with averages over $\lambda_{J_{0}}\equiv 2^{J_{0}}\Delta t$ scale, and
- the scaling filter $\left\{g_{J_{0}}\right\}$ covers the low-frequency range $[0,1/2^{J_{0}+1}]\frac{1}{\Delta t}$ (low frequency and therefore the smoother appearance of $V_{J_{0}}$ and accordingly the associated synthesized signal *A*[*J*_0_ = 5] (see S2 Fig). For first-level relation between *V*_1_ and *A*[1], see S3 Fig central row).

One class of such filters is the Daubechies-extremal phase filter [41] known as the minimum delay filter because at the output, signals build up energy in shortest amount of time compared to other filter realizations [42].

Another important class is the *Least Asymmetric (LA) -filter*. Filter with zero phase function or a linear phase function are best suited for applications where the synthesized (filter output) signals need to be aligned in time with signals at the filter input for better event occurrence (features) interpretation. We can obtain an approximate zero phase filter from shifting *LA* filter output. At higher scales *j*>2, {*g*_*j*_} and {*h*_*j*_} filters have better approximation to zero phase filters if *L*/2 is even, therefore the choice of the *LA*(8) wavelet filter with length *L* = 8 in this study.

Higher *L* values on the other hand ensures that the resulting coefficients after filtering a stochastic process *X* are to be considered *stationary stochastic process realizations* with sample mean equals zero, if *X* is stationary or *L*>2*d* and *X* is *d* backward stationary. Stationarity is crucial assessing statistical properties of a process and a sample mean that is zero ensures an unbiased estimation of the sample variance (for first level MODWT-decomposition and -reconstruction of *X* using *LA*(8) filters see S3 Fig).

Finally, MODWT coefficients {*W*_*j*_} and $\left\{V_{J_{0}}\right\}$ are used for exact scale-based sample variance decomposition. Equation Eq (a) defines the sample variance, while (b) expresses the contribution of each decomposition level (*j*) based on MODWT coefficients to the overall sample variance. From this, it is now possible to identify scales that play a major role in the process variance.

(a) ${\hat{\sigma_{X}}}^{2}=\frac{1}{N}{\left\|X\right\|}^{2}-{\bar{X}}^{2}$ with $\bar{X}$ being the sample mean
(b) ${\hat{\sigma_{X}}}^{2}=\frac{1}{N}\sum_{j=1}^{J_{0}}{\left\|W_{j}\right\|}^{2}+\frac{1}{N}{\left\|V_{J_{0}}\right\|}^{2}-{\bar{X}}^{2}$ note that *j* denotes the level of decomposition.

In case of $N=2^{J_{0}}$ we have $\frac{1}{N}{\left\|V_{J_{0}}\right\|}^{2}-{\bar{X}}^{2}=0$ otherwise, the term represents the sample variance of the smoothed version $V_{J_{0}}$ of the original timeseries *X*.

For the sake of completeness, the sample energy can also be expressed in terms of scale-based decomposition coefficients:

(c) ${\left\|X\right\|}^{2}=\sum_{j=1}^{J_{0}}{\left\|W_{j}\right\|}^{2}+{\left\|V_{J_{0}}\right\|}^{2}$ energy decomposition of *X* up to level *J*_0_.

${\left\|X\right\|}^{2}=\sum_{j=1}^{5}{\left\|W_{j}\right\|}^{2}+{\left\|V_{5}\right\|}^{2}$ is the energy decomposition up to level 5 (for a graphic description of the decomposition levels and corresponding timeseries see S4 Fig).

As noted before, the sample variance of *X* is expressed as the sum of the scale-based sample variances of {*W*_1_} to {*W*_5_} plus the sample variance of {*V*_5_}. Scales that mostly contribute to the overall process variance are also the ones that are more interesting for scientific analysis (see S4 Fig scale 3 for task, scales 4 and 5 for rest).

Although Eq (b) is the exact scale-based sample variance decomposition, it still needs uncertainty estimation of the results. For each decomposition level (*j*), a new quantity referred to as wavelet variance was defined. Wavelet variance *ν*^2^(*τ*_*j*_) computes the scale-based variance of random variables {*W*_*j*,*t*_}, and its corresponding confidence intervals (see S4 Fig right). An unbiased MODWT estimator calculation of wVar was performed. For the uncertainty estimation of this estimator, we followed the recommendations by Percival and Walden [40, p.315] and determined confidence intervals based on *η*_3_ (we chose the conservative approach because timeseries were relatively short). Moreover, it was successfully applied to a wide spectrum of stochastic processes including stationary, *d*^*th*^ order backward difference stationary and nonstationary backward differenced locally stationary processes [30]. WVar was determined in three different frequency bands: 0.02–0.04Hz, 0.04–0.08Hz and 0.08–0.16Hz [27, 28, 43, 44].

### Statistical analysis

To address differences in wVar between task- and rs-fMRI, 2x2 MANOVA models were defined with the factors *group* (ADHD vs. TDC) and *fMRI condition* (task vs. rest) and ROI-and scale-specific wVar coefficients as dependent variables. Within the MANOVA workflow, post-hoc t-tests were included to identify significant effects between subgroups. The influence of cognitive load on wVar was examined using repeated measures ANOVA models, with the within-subject factor *cognitive load* (block 1 vs. block 3 vs. block 4 vs. block 5, Note. Block 2 was a baseline block without reward), the between-subject factor *group* (ADHD vs. TDC) and ROI-and scale-specific wVar coefficients/behavioral performance measures (i.e., premature responses, accuracy, reaction times) as dependent variables. Within the ANOVA workflow, post-hoc t-tests were integrated to identify significant effects between conditions. In a final step, behavioral performance was directly related to wVar via group-specific bivariate correlations. To test for statistical significance of correlation coefficients (R_ADHD_ vs. R_TDC_), fisher-r-to-z-transformations were calculated.

The influence of the *TPH2 G-703T* polymorphism was addressed via 2x2 MANOVA models with the factors *group* (ADHD vs. TDC) and *TPH2 G-703T* variant (GG vs. T^+^) and ROI-and scale-specific wVar coefficients/behavioral parameters as dependent variables. Post-hoc t-tests were performed to identify significant differences between subgroups. To address the effect of DNA methylation of 6 CpG sites were calculated using linear multiple regression analyses. As potential confounding variables age, sex and the *TPH2 G-703T* variation entered in the first block (inclusion) and the remaining variables competed in the second block for inclusion by using the stepwise algorithm. Dependent variables were ROI-and scale-specific wVar coefficients/behavioral parameters.

In all MANOVA-models, sex and age entered as nuisance variables and results of all analyses were reported on p < .05, corrected for multiple comparisons using false discovery rate (FDR) correction. Additionally, post-hoc power analyses were performed using partial eta squared with small effect size = 0.01; medium effect size = 0.06; large effect size = 0.14.

## Results and discussion

In both groups, wVar coefficients did not vary in function of age nor differed between sexes. The same was true for epigenetics; DNA methylation did not differ significantly between sexes and was not correlated with age, neither in TDC nor in ADHD patients. Additionally, methylation levels did not differ significantly between ADHD and TDC (S1 Table).

The 2x2 MANOVA Models revealed significant *main effects of condition* in the rs-fMRI-associated low frequencies 0.02–0.04Hz in the FPN.r.LPFC with wVar being higher at rest compared to task (no significant group effects). In return, significant *group* X *condition interactions* were found in the cognitive-related high frequencies of 0.08–0.16Hz in medial and lateral PFC regions of both networks (DMN.MPFC, FPN.l.LPFC, FPN.r.LPFC). WVar in TDC decreased from rest to task, whereas ADHD patients showed a reverse pattern with higher wVar at task compared to rest (see Table 1).

**Table 1:** Significant results of 2x2 MANOVA models using *condition* and *group* and ROI-and scale-specific wVar.

	ADHD_task_	ADHD_rest_	TDC_task_	TDC_rest_	F_group_	F_cond_	F_groupXcond_	p_η^2^
	[M(SD)]	[M(SD)]	[M(SD)]	[M(SD)]				
**Scale 3**								
DMN.MPFC	0.052(.04)	0.044(.04)	0.039(.02)	0.067(.05)	0.5	2.0	6.7*	.063
FPN.l.LPFC	0.041(.03)	0.027(.01)	0.030(.02)	0.056(.05)	2.9	1.2	12.9*	.095
FPN.r.LPFC	0.047(.03)	0.034(.02)	0.034(.02)	0.056(.05)	0.6	0.8	9.4*	.073
**scale 5**								
FPN.r.LPFC	0.040(.02)	0.029(.02)	0.037(.04)	0.062(.06)	4.9	6.9*	1.2	0.75

Note. DMN: default mode network, DMN.MPFC: medial prefrontal cortex; FPN: fronto-parietal network, FPN.r.LPFC/FPN.l.LPFC: right and left lateral PFC; frequency bands: scale 3 = 0.08–0.16Hz, scale 5 = 0.02–0.041Hz.

*: significant with p_FDR_<q = .011; p_eta^2^: partial eta squared with small effect size = 0.01; medium effect size = 0.06; large effect size = 0.14.

**Fig 2:**
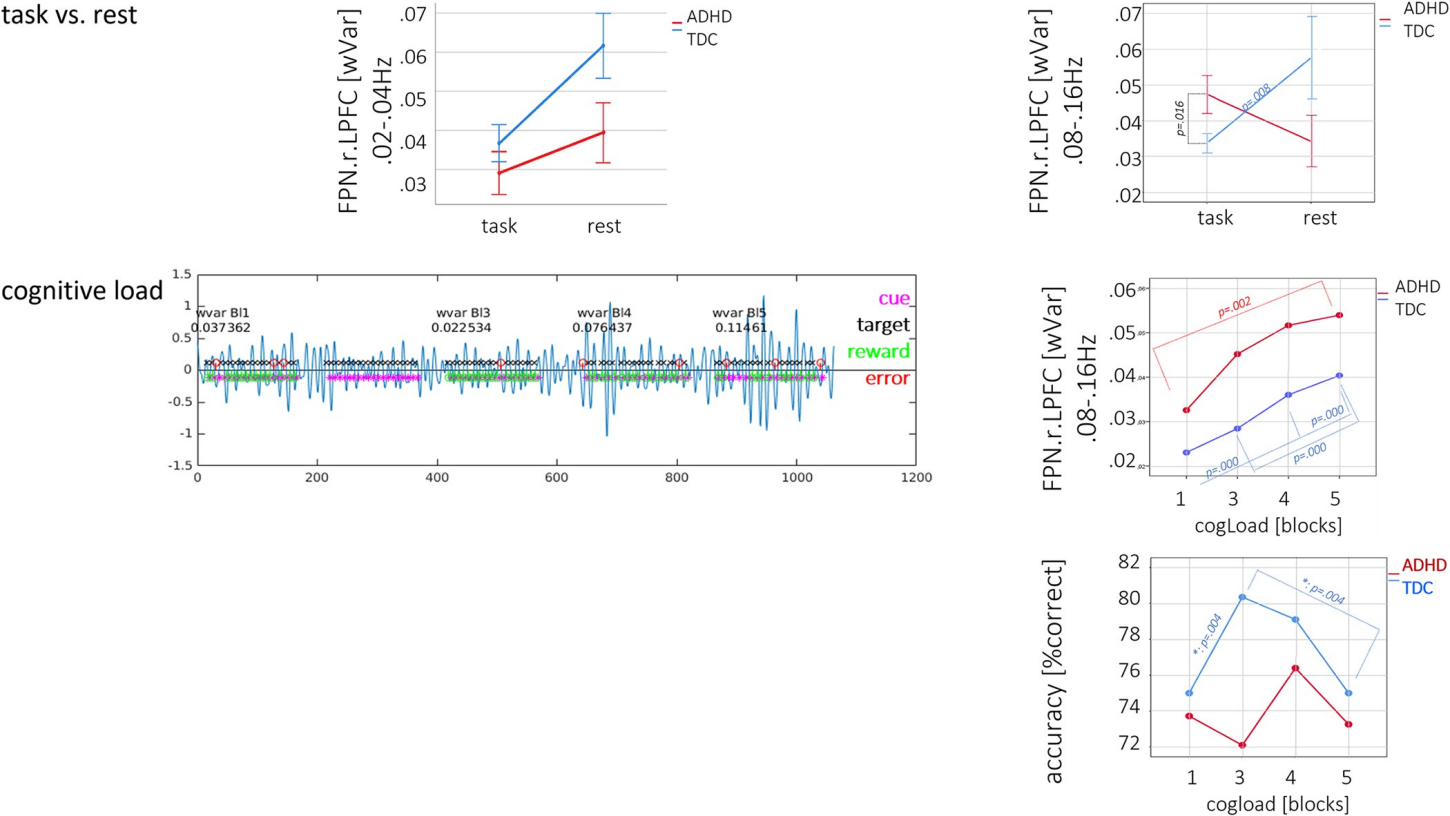
Brain dynamics as measured using wVar. Fig 2 presents differences of wVar between rs-fMRI and task-fMRI (upper row) and the influence of cognitive load on wVar (lower row) at the example of FPN.r.LPFC. Error bars represent 1SE.

Post-hoc t-tests revealed that wVar in the high frequencies of FPN.r.LPFC under task was significantly higher in ADHD compared to TDC (T = 2.5, p = .016) and in TDC, wVar in all three regions was higher at rest compared to task in the cognitive frequencies (DMN.MPFC: T = 3.1, p = .003, FPN.l.LPFC: T = 3.3, p = .002, FPN.r.LPFC: T = 3.0, p = .004) and uncorrected also in the low frequencies 0.02–0.04Hz (FPN.r.LPFC: T = 2.2, p = .034) (Fig 2). To rule out, that differences in wVar between groups were based on differences in brain activation, small volume corrected analyses (i.e., 10mm spheres around the regional maxima) of brain activation maps were performed showing no overlap between group effects of wVar and neural activation (see S2 Table). Across both groups, wVar increased significantly with cognitive load in the cognitive frequencies of FPN.r.LPFC; however, wVar was significantly higher in ADHD patients compared to TDC across all blocks (Table 2). On the behavioural level, a U-shaped effect in accuracy in TDC was revealed with an increase from block 1 to 3, followed by a significant decrease in the last two blocks (Fig 2). In ADHD patients, there was no significant effect of task difficulty and no difference in accuracy across all blocks (S3 Table). Finally, wVar significantly correlated with accuracy and number of errors in cognitive frequencies of FPN.r.LPFC in ADHD patients but not TDC (accuracy: R_ADHD_ = -.388, p = .010, R_TDC_ = -.053, p = .699. Z = 1.7, p = .044; # of errors (excl. misses): R_ADHD_ = .371, p = .014, R_TDC_ = .075, p = .583. Z = 1.5, p = .067).

**Table 2:** Significant results of 2x4 repeated measures MANOVA models using *cognitive load* and *group*, and wVar.

ROI	block	ADHD	TDC	F_cogLoad_	F_group_	F_int_	p-η^2^	p-η^2^
		[M(SD)]	[M(SD)]				[cogLoad]	[group]
**Scale 3**								
FPN.r.LPFC	block1	.03(.02)	.02(.01)	22.7*	5.1*	0.2	.190	0.05
	block3	.05(.04)	.03(.02)					
	block4	.05(.05)	.04(.03)					
	block5	.05(.05)	.04(.02)					

**Note.** FPN: fronto-parietal network, FPN.r.LPFC: right lateral PFC; frequency bands: scale 3 = 0.08–0.16Hz, scale 5 = 0.02–0.041Hz.

*: significant with p_FDR_< q* = .004; p-η^2^: partial eta squared with small effect size = 0.01; medium effect size = 0.06; large effect size = 0.14.

*TPH2 G-703T* MANOVA models on behavioral performance revealed significant group differences in reaction times (F_group_ = 8.5, p = .005, F_TPH2_ = 1.0, n.s.; F_int_ = 0,1, n.s.). Post-hoc t-tests showed that only the T-allele carriers significantly differed between groups with ADHD T^+^ presenting the longest reaction times (GG_ADHD_ = 467(96), GG_TDC_ = 415(46), T^+^_ADHD_ = 486(90), T^+^_TDC_ = 433(51), GG_TDC_<GG_ADHD_: T_rt_ = 2.0, p = .056, GG_TDC_<T^+^_ADHD_: T_rt_ = 2.8, p = .009; T^+^_TDC_<T^+^_ADHD_: T_rt_ = 2.2, p = .041).

**Fig 3:**
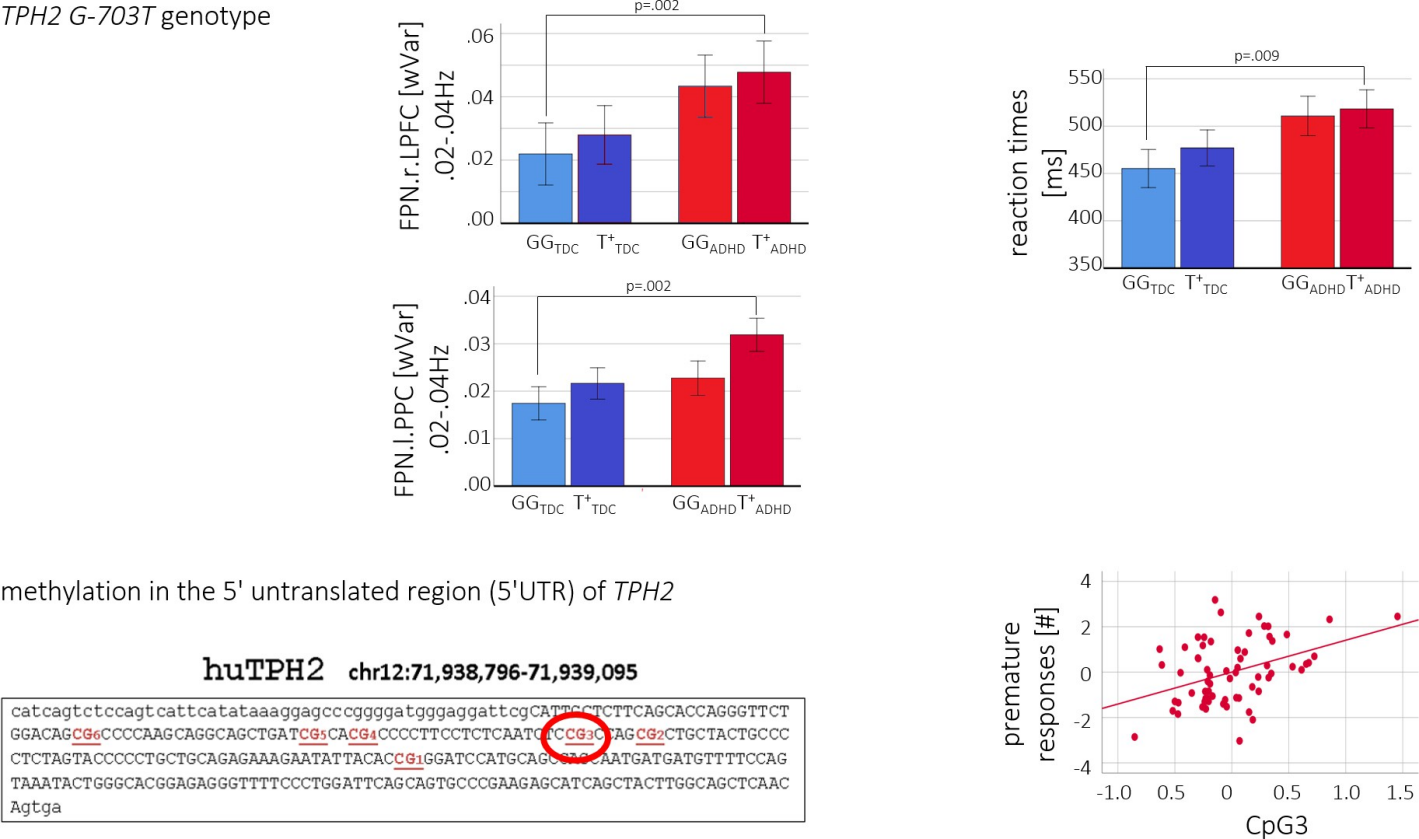
Serotonergic modulation. Fig 3 shows the results of the *TPH2 G-703T* variants on FPN.r.LPFC wVar and behavioural performance (upper row) as well as methylation on behavioural performance (lower row).

On the neural level, similar patterns were found in the low frequencies 0.02–0.04Hz of the right hemispheric FPN network regions (i.e. FPN.r.LPFC, FPN.r.PPC) and in the cognitive frequencies in DMN.l.LP. Post-hoc t-tests revealed (trend to) significant differences between GG_TDC_ and T^+^_ADHD_ (GG_TDC_<T^+^_ADHD_: T_FPN.r.LPFC_ = 3.5, p = .002; T_FPN.r.PPC_ = 3.4, p = .002; T_DMN.l.LP_ = 2.2, p = .034) as well as T-allele carriers of both groups (T^+^_TDC_<T^+^_ADHD_: T_FPN.r.LPFC_ = 2.2, p = .036; T_FPN.r.PPC_ = 2.0, p = .057; T_DMN.l.LP_ = 1.8, n.s.) with highest variance in ADHD T^+^ (see Table 3 and Fig 3).

**Table 3:** Significant results of 2x2 MANOVA models using *TPH2 genotype* and *group*, and ROI- and scale-specific wVar.

	GG_ADHD_	T^+^_ADHD_	GG_TDC_	T^+^_TDC_	F_TPH2_	F_group_	F_int_	p-η^2^
	[M(SD)]	[M(SD)]	[M(SD)]	[M(SD)]				[group]
**scale 3**								
DMN.l.LP	.03(.02)	.03(.02)	.02(.01)	.03(.01)	n.s.	5.7*	n.s.	.087
**scale 5**								
FPN.r.LPFC	.04(.07)	.05(.03)	.02(.01)	.03(.02)	n.s.	4.5*	n.s.	.069
FPN.r.PPC	.02(.02)	.03(.02)	.02(.01)	.02(.01)	n.s.	5.4*	n.s.	.082

**Note.** DMN: default mode network, DMN.LP: lateral parietal cortex; FPN: fronto-parietal network, FPN.r.LPFC: right lateral PFC, FPN.r.PPC: right posterior parietal cortex; frequency bands: scale 3 = 0.08–0.16Hz, scale 5 = 0.02–0.041Hz. *: significant with p_FDR_<q* = .033; η^2^: partial eta squared with small effect size = 0.01; medium effect size = 0.06; large effect size = 0.14.

CpG3 methylation significantly predicted behavioral as well as wVar coefficients, however, in ADHD patients but not TDC. On the behavioral level, CpG3 methylation predicted premature responses (F_model_ = 3.7, R^2^ = 0.234). On the neural level, multiple regressions revealed a significant influence of CpG3 methylation and low frequency fluctuations in right FPN regions (i.e. FPN.l.LPFC: F_model_ = 3.3, R^2^ = 0.397 and FPN.l.PPC: F_model_ = 4.4, R^2^ = 0.405) with higher methylation/lower serotonin being correlated with higher wVar (see Table 4 and Fig 3). Post-hoc power analyses using G*Power determined for an assumed low to moderate effect size (H1 p^2^ = 0.23) a power of 0.85 and a critical R^2^ = 0.234.

**Table 4:** Prediction of WI (ROI- and scale-specific wVar and behavioral performance): Results of the multiple stepwise regression analysis.

Model		B	Std	Beta	T	Sig.
Premature Responses						
1	(Constant)	4.304	1.523		2.8	.006
	TPH2	-.654	.388	-.214	-1.7	.096
	age	-.090	.079	-.147	-1.1	.262
	sex	.182	.539	.043	0.3	.737
2	(Constant)	4.926	1.479		3.3	.001
	TPH2	-.576	.373	-.189	-1.5	.127
	age	-.016	.082	-.026	-0.2	.845
	sex	.219	.517	.051	0.4	.674
	CpG3	1.088	.418	.363	2.6*	.012
FPN.l.LPFC scale5						
1	(Constant)	.029	.035		0.8	.404
	TPH2	.006	.008	.139	0.7	.471
	Age	-4.418^E-5^	.002	-.005	-0.1	.982
	Sex	-.013	.012	-.220	-1.1	.292
2	(Constant)	.022	.032		0.7	.500
	TPH2	.002	.007	.058	.3	.744
	age	-.003	.002	-.294	-1.4	.183
	sex	-.019	.011	-.321	-1.7	.103
	CpG3	.021	.008	.522	2.6*	.015
FPN.l.PPC_scale5						
1	(Constant)	.092	.034		2.7	.012
	TPH2	.013	.007	.301	1.8	.087
	age	-.004	.002	-.403	-2.3	.034
	sex	-.023	.012	-.357	-2.0	.061
2	(Constant)	.084	.031		2.7	.012
	TPH2	.010	.007	.229	1.5	.155
	age	-.007	.002	-.659	-3.5*	.002
	sex	-.029	.011	-.446	-2.6*	.014
	CpG3	.020	.008	.463	2.6*	.015

**Note.** FPN: fronto-parietal network, FPN.l.LPFC: left lateral PFC, FPN.l.PPC: left posterior parietal cortex; frequency band: scale 5 = 0.02–0.041Hz. *: significant with p_FDR_<q* = .015. Post-Hoc power = 0.85, critical R^2^ = 0.234.

Across all analyses, there was any significant effect in scale 4, the (intermediate) frequencies 0.04–0.08Hz.

To check for a potential influence of comorbid affective disorders on genetic effects, MANOVA models were performed with affective comorbidity as covariate of interest showing no significant influence (see S4 Table).

For an external validation of group comparisons in wVar at rest, we determined wVar in rs-fMRI timeseries of a subsample of the Child Mind Institute data set (Functional Connectomes Project International Neuroimaging Data-Sharing Initiative http://dx.doi.org/10.15387/CMI_HBN (2017)). The Child Mind Institute has launched the Healthy Brain Network, with participants aged from 5–21yrs including psychiatric phenotypes and multimodal brain imaging (e.g., rs-fMRI, morphometric MRI). Based on the specificity of the used task and genetic data, we were only able to validate comparisons of wVar at rest between ADHD patient (N = 124) and control subjects (i.e., ‘no diagnosis given’, N = 126). Results replicated our findings in terms of no significant groups differences in wVar at rest in combination with similar or lower absolute values in ADHD patients compared to control subjects (see S5 Table).

In line with our hypotheses, we found that in TDC/normal processing, wVar in the cognitive frequencies of the FPN.LPFC was significantly higher at rest compared to task. In the recent years of research on resting-state networks [45, 46], the frequently used terms to describe resting-state neural activity were “intrinsic”, “endogenous,” and “spontaneous,” indicating that resting-state network function is produced within the brain itself and can, thus, be understood as “self-organized” [47]. In this case, a task-induced stimulation can be understood as an intervention from the external environment leading to a reduction/rearrangement of these dynamics. Empirical evidence using wVar in this context is lacking to date, however earlier studies have shown higher fMRI variance at rest compared to task before. For example, He et al (2011) showed a significant decrease in fMRI signal variance during a visual detection task compared to rs-fMRI in resting state networks such as default and attention networks [48], i.e., in networks, where also wVar showed a significant condition effect in our analysis. Thus, findings of the current study can be interpreted in this line of argumentation and extend earlier findings by showing that this pattern was disturbed in pathological brain function in the example of ADHD.

Abnormal processing in ADHD, in return, was characterized by higher wVar under task compared to rest in both networks and across cognitive and rs-associated frequencies. According to the *default mode interference* hypothesis, “generalized task-non-specific cognition during rest can persist or intrude into periods of active task-specific processing, producing periodic fluctuations in attention that compete with goal-directed activity” [34]. Thus, higher wVar in ADHD patients in the cognitive frequencies of DMN.MPFC during task processing might reflect the described impaired DMN suppression [32], and interaction between task-specific (FPN) and unspecific (DMN) activation [34]. When interpreting higher wVar during task in ADHD patients not as ‘moment-to-moment variability of spontaneous brain signals’ but rather in terms of attempts to adjust or fine-tune brain function to complete the task (i.e. processes of self-organization), findings of higher wVar in the cognitive frequencies of the FPN.r.LPFC, might reflect the intense concentration on the task and/or coping with task difficulty, as it significantly increased with cognitive load and was correlated with performance accuracy. Coping with increasing task difficulty have been reported in terms of individuals disengaging when they reached their limits [49], or shifting strategies, e.g. the recruitment of different brain regions [50]. Load-induced increase in wVar, on the other hand, revealed that ADHD patients neither seemed to shift their strategy nor to disengage completely, but instead kept changing their modulation attempts within the same region to perform the task, resulting in significantly higher wVar in ADHD patients compared to TDC across all blocks. Interestingly, we furthermore found that only in TDC these modulations did indeed lead to the expected U-shaped behavioural performance (see Fig 2 right side line plots). Thus, an increase in wVar in ADHD patients did not necessarily result in the maintenance of behavioural performance but rather reflected the effort to do so [51].

Interpreting the dynamics we found in our study from a signal processing perspective, wVar is rather specific to input than outcome as it varies in function of cognitive load and not behavioral performance. As soon as task difficulty changes, the block-specific task (input) demands a new signalling pattern from the target region (in our case the FPN.r.LPFC), e.g., in terms of shifting strategies. This increase in cognitive processing is reflected by the increase in wVar. What differentiates normal from abnormal brain dynamics is, that these dynamics result in predictable outcome (i.e., an inverted U-shaped curve of behavioral performance) in TDC and a random curve in behavioral performance in ADHD patients. This signal processing interpretation, thus, links the two assumptions regarding the influence of cognitive load (disengage or shift of region) together, namely, in normal processing, it can only come to a disengagement of activation or the recruitment of further regions after the assigned region has been able to respond to the challenge with an appropriate signalling pattern. However, ADHD patients seemed to be unable to use neither one of these strategies as no adequate signalling pattern was found to cope with increasing task difficulty. Thus, we can summarize, that wVar was able to detected even immediate changes in network dynamics during WI processing and that it is a highly sensitive marker towards ADHD-specific alteration in brain dynamics.

Finally, correlations between wVar and behavioral performance were only significant in ADHD but not in TDC, which might be related to the ongoing attempt of the task-stimulated region to adapt to the new demands in ADHD patients while in TDC such adaptation was not necessary. The most robust findings of highly variable processing in ADHD have been published on reaction times, where ADHD patients showed higher individual variability compared to TDC [52]. A meta-analysis by Kofler et al. (2013) revealed that children and adolescents (Hedges’ g = 0.76) and adults (g = 0.46) with ADHD demonstrated greater variability relative to control groups (results were corrected for measurements of unreliability and publication bias) [16]. However, recent studies also introduced dynamic functional connectivity as a promising biomarker in the context of ADHD, suggesting new parameters to quantify the dynamics within and between networks such as recruitment rate, topology of specific synergies between resting-state networks and synergetic cooperation patterns [24] as well as using the sliding window approach in terms of time-varying covariance of interregional neural signals [53]. A wavelet-based parameter like wVar, thus, adds valuable information to the current scientific debate to rather focus on dynamics in fMRI than static parameters in ADHD patients. Furthermore, wVar can be determined in both, fMRI timeseries at rest and under task and can, therefore, be directly linked to cognitive processes via behavioral performance. Lacking relation between performance and wVar in TDC might be explained by the fact, that wVar is supposed to reflect input-induced processes and is not necessarily related to behavioral outcome. Therefore, wVar in ADHD patients with good performance was also low, similar to TDC. In patients with poor performance, in return, wVar increased indicating ongoing attempts to find the optimal modulation of ROI-specific signaling.

The (epi)genetic influence was found predominantly in the rs-fMRI-associated frequencies in regions of the FPN. The lateral PFC has robustly been reported being part of the serotonergic system, in the rodent [54] as well as the human brain [55, 56]. That mainly low frequencies, i.e., basic neural functioning, were modulated hints towards a serotonergic influence on a very general level and independent of cognitive processing and/or external stimulation. In line with earlier findings, we found a gene-dosage effect with highest wVar and longest reaction times in ADHD T-allele carriers (pathology + reduced serotonin) [57]. Interestingly, there are some hints towards the G-allele being associated with ADHD. For example, higher transmission frequencies of the G-allele were shown in ADHD families [58] and decreased PFC activation during response inhibition was reported in ADHD patients with the rs4570625 GG genotype in an EEG-study [55]. Ambiguous findings might support the introduced indirect effect of serotonin in the context of ADHD and vary in terms of the dependent variables. For example, in this study, we quantified not brain activation/functional connectivity like Baehne et al. (2009) but ADHD-relevant variability, which might be more sensitive to serotonin. This assumption is supported by the significant gene-dosage effect in reaction times. As reaction time variability was the first dimension, where an increased variability in ADHD patients has been documented [52], it would be interesting to replicate serotonergic modulation of ‘neural/behavioral variability’ also in the context of affective disorders, where serotonin has been proven to play a significant role [59].

Finally, an ADHD-specific positive linear effect between CpG3 methylation and fronto-parietal wVar and impulsivity/premature responses was found. An ADHD-specific relation between DNA methylation and reaction time variability in a motivational Go/NogoTask has been reported before [13], supporting the influence of DNA methylation on both, variability and impulsivity. DNA methylation has been linked to personality traits/endophenotypes such as aggression [60], and the ability to recognize mental states [38] as well as in the context of affective disorders [12, 61] to rs-fMRI-related PFC activity [62].

## Conclusions

The candidate gene approach has been challenged in the context of the genetic basis of neuropsychiatric disorders. Thus, the use of (epi)genetic variation in a single gene in this study should be understood as a methodological approach to go deeper into the mechanism of how genotype and DNA methylation manifests itself in the brain. However, to combine both approaches, e.g., reporting an EWAS in clinical samples [35] with subsequent regression analyses between DNA methylation and representative endophenotypes would be of high interest in future studies. In this paper, we stated that the influence of serotonin in ADHD might be stronger in those patients with comorbid depression and/or anxiety. Therefore, we performed genetic analyses with additional ‘comorbid with affective disorder’ covariate. There was no effect in any analysis, strengthening our interpretation that serotonin modulates the FPN and impulsivity on a very basic level. Finally, we found that methylation effects were only at CpG3, however, on the basis of the earlier findings, a functional interpretation remains speculation. Therefore, we decided to postpone this to future studies, replicating or extending the current findings.

In sum, in this study, we were able to show that wVar is a sensitive marker towards altered neural processing in ADHD and that both, the genotype and methylation levels of the *TPH2 G-703T* polymorphism modulate the same on the neural and behavioral level.

## Supporting information

S1 Fig The four choice serial reaction time task. On the left side an exemplary trial is presented. On the right side, block-specific task difficulty manipulation is depicted.
(TIF)

S2 Fig Scale-based decomposition. S2 Fig presents an overview of the relationship between decomposition levels (*j*) up to level (*J*_0_ = 5), timescales *τ*_*j*_ and corresponding frequency bands in case of fMRI timeseries *Δt* = 0.8*sec*. X = D1+D2+D3+D4+D5+A5 multiresolution analysis of X. **Note.** *D*[*j*] and *A*[*J*_0_] are defined in S3 Fig., *: definition of λ_5_ and its corresponding frequency band, see 2.5.
(TIF)

S3 Fig Decomposition filters. S3 Fig shows *LA*(8)-filter applied to *X* at level 1, decomposition filters (dec) compute the wavelet and the scaling coefficients *W*_1_ and *V*_1_. At the output of the reconstruction filters (rec), *D*_1_ and *A*_1_ are the zero-phase synthesized signals, representing the high and low frequency portion (**D**etail and **A**pproximation, respectively) of the signal *X*. dec HF: *LA*(8) decomposition high-pass filter (dec HF) coefficients: {-0.0322, -0.0126, 0.0992, 0.2979, -0.8037, 0.4976, 0.0296, 0.0758} *LA*(*8*) decomposition low-pass filter (dec LF) coefficients: {-0.0758, -0.0296, 0.4976, 0.8037, 0.2979, -0.0992, -0.0126, 0.0322} *LA*(8) reconstruction high-pass filter (rec HF) coefficients: {-0.0758, 0.0296, 0.4976, -0.8037, 0.2979, 0.0992, -0.0126, -0.0322} *LA*(8) reconstruction low-pass filter (rec LF) coefficients: {0.0322, -0.0126, -0.0992, 0.2979, 0.8037, 0.4976, -0.0296, -0.07577}.
(TIF)

S4 Fig Multiresolutional analysis of fMRI timeseries. S4 Fig left shows condition-specific multiresolution analysis of exemplary fMRI timeseries. During task, higher frequencies (lower scales) contribute the most to the overall fluctuation and variance of the signal. At rest, lower frequencies (higher scales) are the dominant contributors. Right, wVar and its corresponding CI are plotted. Dots represent the wVar for task (blue) and rest (green), lines indicate CI intervals (blue dashed = task, green solid = rest). For the wavelet variance estimation, there are fewer data points at rest compared to task (scale1: 443 vs. 1056, scale2: 429 vs. 1042, scale3: 401 vs. 1014, scale4: 345 vs. 958, scale5: 233 vs. 846), hence wider Cis.
(TIF)

S1 Table Sample description. ADHD: Attention Deficit/Hyperactivity Disorder, TDC: typically developing children, IQ: intelligence quotient, M = Mean, SD = standard deviation, Accuracy = ((misses+errors)/total number trials)*100; CpG: cytosine–phosphate–guanine site;*: significant with p_FDR_<q = .011.
(DOCX)

S2 Table Group differences between ADHD patients and TDC in ROI activation. DMN: default mode network, DMN.MPFC: medial prefrontal cortex, DMN.LP: lateral parietal cortex, DMN.PCC: posterior cingulate cortex; FPN: fronto-parietal network, FPN.LPFC: lateral PFC, FPN.PPC: posterior parietal cortex.
(DOCX)

S3 Table Effects of cognitive load on behavioral performance. ADHD: Attention Deficit/Hyperactivity Disorder, TDC: typically developing children, cogLoad: cognitive load, scale 3 = 0.0781–0.1562Hz. *: significant with p_FDR_ < .004.
(DOCX)

S4 Table Significant results of 2x2 MANCOVA models. DMN: default mode network, DMN.LP: lateral parietal cortex; FPN: fronto-parietal network, FPN.r.LPFC: right lateral PFC, FPN.r.PPC: right posterior parietal cortex; frequency bands: scale 3 = 0.08–0.16Hz, scale 5 = 0.02–0.041Hz. *: significant with p_FDR_<q* = .033; η^2^: partial eta squared with small effect size = 0.01; medium effect size = 0.06; large effect size = 0.14.
(DOCX)

S5 Table External validation results of univariate ANOVA models using *group* as independent factor and ROI-and scale-specific wVar from rs-fMRI timeseries only as dependent variable (ADHD: N = 124, no diagnosis given: N = 145). **Note.** DMN: default mode network, DMN.MPFC: medial prefrontal cortex; FPN: fronto-parietal network, FPN.r.LPFC/FPN.l.LPFC: right and left lateral PFC; frequency bands: scale 3 = 0.08–0.16Hz, scale 5 = 0.02–0.041Hz. p_eta^2^: partial eta squared with small effect size = 0.01; medium effect size = 0.06; large effect size = 0.14.
(DOCX)

S1 Methods (DOCX)
