# Supplementary figures and images for: Serotonergic modulation of normal and abnormal brain dynamics: The genetic influence of the TPH2 G-703T genotype and DNA methylation on wavelet variance in children and adolescents with and without ADHD
Source: PLoS One. 2023 Apr 27;18(4):e0282813. doi: 10.1371/journal.pone.0282813 (PMC10138254; doi:10.1371/journal.pone.0282813)

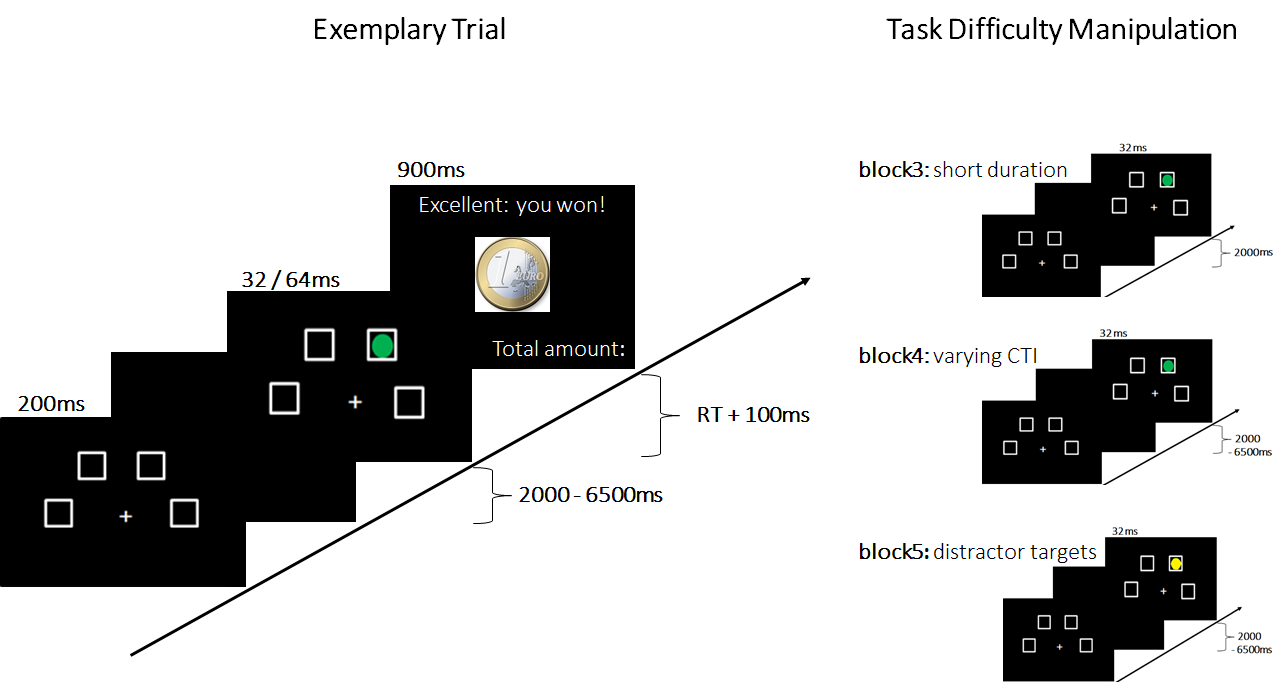

Supplement: S1 Fig — On the left side an exemplary trial is presented. On the right side, block-specific task difficulty manipulation is depicted. (TIF) [file pone.0282813.s001.tif]

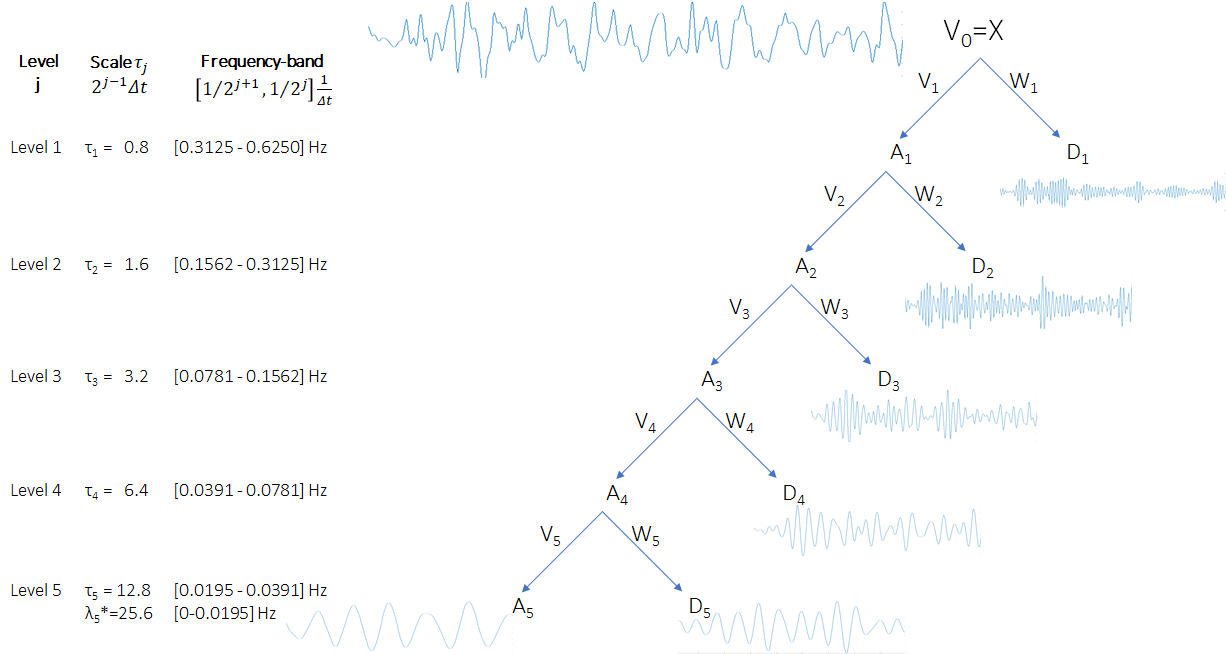

Supplement: S2 Fig — presents an overview of the relationship between decomposition levels (j) up to level (J0 = 5), timescales τj and corresponding frequency bands in case of fMRI timeseries Δt = 0.8sec. X = D1+D2+D3+D4+D5+A5 multiresolution analysis of X. Note. D[j] and A[J0] are defined in S3 Fig., *: definition of λ5 and its corresponding frequency band, see 2.5. (TIF) [file pone.0282813.s002.tif]

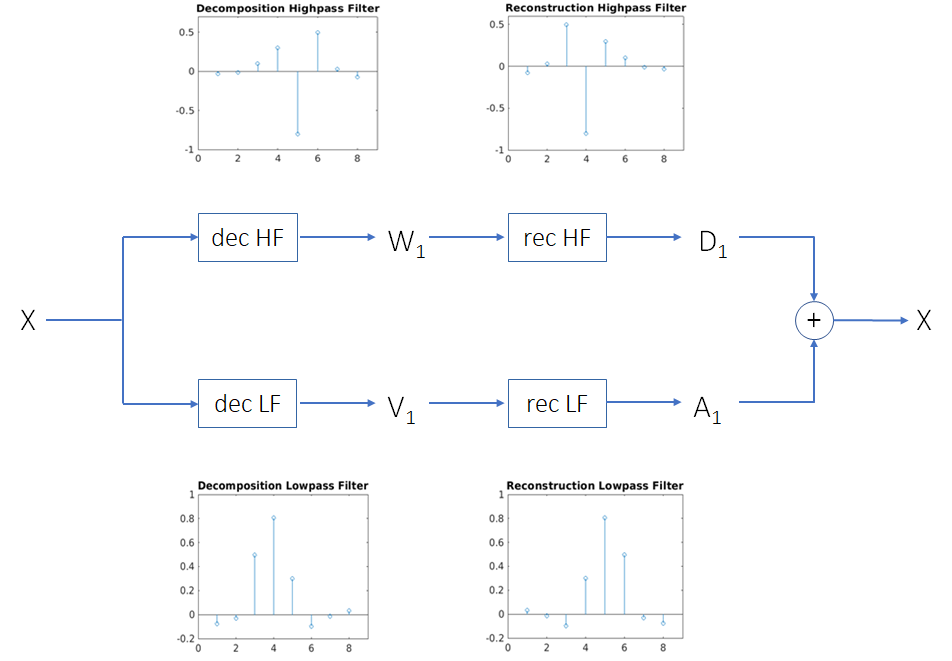

Supplement: S3 Fig — shows LA(8)-filter applied to X at level 1, decomposition filters (dec) compute the wavelet and the scaling coefficients W1 and V1. At the output of the reconstruction filters (rec), D1 and A1 are the zero-phase synthesized signals, representing the high and low frequency portion (Detail and Approximation, respectively) of the signal X. dec HF: LA(8) decomposition high-pass filter (dec HF) coefficients: {-0.0322, -0.0126, 0.0992, 0.2979, -0.8037, 0.4976, 0.0296, 0.0758} LA(8) decomposition low-pass filter (dec LF) coefficients: {-0.0758, -0.0296, 0.4976, 0.8037, 0.2979, -0.0992, -0.0126, 0.0322} LA(8) reconstruction high-pass filter (rec HF) coefficients: {-0.0758, 0.0296, 0.4976, -0.8037, 0.2979, 0.0992, -0.0126, -0.0322} LA(8) reconstruction low-pass filter (rec LF) coefficients: {0.0322, -0.0126, -0.0992, 0.2979, 0.8037, 0.4976, -0.0296, -0.07577}. (TIF) [file pone.0282813.s003.tif]

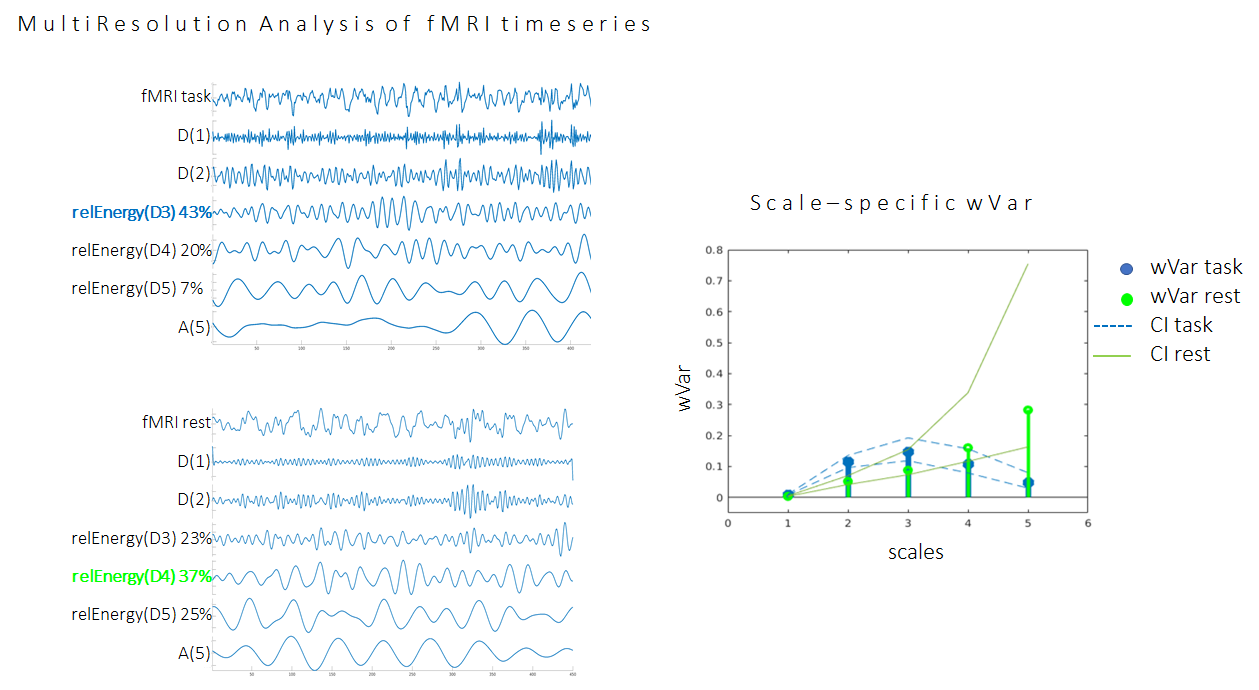

Supplement: S4 Fig — left shows condition-specific multiresolution analysis of exemplary fMRI timeseries. During task, higher frequencies (lower scales) contribute the most to the overall fluctuation and variance of the signal. At rest, lower frequencies (higher scales) are the dominant contributors. Right, wVar and its corresponding CI are plotted. Dots represent the wVar for task (blue) and rest (green), lines indicate CI intervals (blue dashed = task, green solid = rest). For the wavelet variance estimation, there are fewer data points at rest compared to task (scale1: 443 vs. 1056, scale2: 429 vs. 1042, scale3: 401 vs. 1014, scale4: 345 vs. 958, scale5: 233 vs. 846), hence wider Cis. (TIF) [file pone.0282813.s004.tif]
