# Supplementary material for: Serotonergic modulation of normal and abnormal brain dynamics: The genetic influence of the TPH2 G-703T genotype and DNA methylation on wavelet variance in children and adolescents with and without ADHD
Source: PLoS One. 2023 Apr 27;18(4):e0282813. doi: 10.1371/journal.pone.0282813 (PMC10138254; doi:10.1371/journal.pone.0282813)
Supplement: S1 Table — ADHD: Attention Deficit/Hyperactivity Disorder, TDC: typically developing children, IQ: intelligence quotient, M = Mean, SD = standard deviation, Accuracy = ((misses+errors)/total number trials)*100; CpG: cytosine–phosphate–guanine site;*: significant with pFDR<q = .011. (DOCX) [file pone.0282813.s005.docx]

**Table s1.** sample description

|  | **ADHD** [M(SD] | **TDC** [M(SD] | **Stats** |  |
| --- | --- | --- | --- | --- |
| N | 64 | 74 |  |  |
| Sex (m/f) | 56/8 | 68/8 | *Χ*^2^=0.7, n.s. |  |
| Age [ | 12.6(2.2) | 12.2(2.4) | T=0.9, n.s. |  |
| IQ [M(SD] | 102.9(13.2) | 108.6(16.1) | T=2.0, n.s. |  |
| **ADHD** **symptoms** |  |  |  |  |
| FBB_ADHD [inattention] | 1.8±0.6 | 0.5±0.6 | T=11.1, p=.000 |  |
| FBB_ADHD [hyperactivity] | 1.3±0.8 | 0.2±0.3 | T=9.2, p=.000 |  |
| CBCL [total] | 45.9±23.9 | 11.4±10.1 | T=9.0, p=.000 |  |
| CBCL [internal problems] | 9.4±7.1 | 4.0±4.0 | T=4.6, p=.000 |  |
| CBCL [external problems] | 15.0±10.0 | 3.7±4.9 | T=6.9, p=.000 |  |
| **Behavioral performance** | | | | |
| premature responses [#] | 2.3(1.4) | 2.4(1.6) | T=0.5, n.s. |  |
| misses [#] | 1.8(3.7) | 1.6(2.0) | T=0.7, n.s. |  |
| errors [#] | 18.3(10.7) | 15.4(12.9) | T=1.0, n.s. |  |
| accuracy [% correct trials] | 76.4(13.9) | 80.6(16.7) | T=1.0, n.s. |  |
| reaction times [ms] | 477(92) | 424(49) | T=2.9, p=.011 |  |
| **Methylation** | | | | |
| CpG1 [%] | 2.5(0.3) | 2.5(0.5) | T=0.3, n.s. |  |
| CpG2 [%] | 3.2(0.8) | 3.0(0.6) | T=1.1, n.s. |  |
| CpG3 [%] | 2.7(0.5) | 2.7(0.5) | T=0.1, n.s. |  |
| CpG4 [%] | 2.0(0.7) | 1.8(0.5) | T=1.6, n.s. |  |
| CpG5 [%] | 2.9(0.8) | 2.7(0.8) | T=1.2, n.s. |  |
| CpG6 [%] | 2.5(0.4) | 2.5(0.5) | T=0.3, n.s. |  |

**Note.** ADHD: Attention Deficit/Hyperactivity Disorder, TDC: typically developing children, IQ: intelligence quotient, M=Mean, SD=standard deviation, Accuracy=((misses+errors)/total number trials)*100; CpG: cytosine–phosphate–guanine site;*: significant with p_FDR_<q=.011.
