# Supplementary material for: Serotonergic modulation of normal and abnormal brain dynamics: The genetic influence of the TPH2 G-703T genotype and DNA methylation on wavelet variance in children and adolescents with and without ADHD
Source: PLoS One. 2023 Apr 27;18(4):e0282813. doi: 10.1371/journal.pone.0282813 (PMC10138254; doi:10.1371/journal.pone.0282813)
Supplement: S2 Table — DMN: default mode network, DMN.MPFC: medial prefrontal cortex, DMN.LP: lateral parietal cortex, DMN.PCC: posterior cingulate cortex; FPN: fronto-parietal network, FPN.LPFC: lateral PFC, FPN.PPC: posterior parietal cortex. (DOCX) [file pone.0282813.s006.docx]

**Table s2** group differences between ADHD patients and TDC in ROI activation *during task processing* (contrast: all vs. baseline) as revealed using small volume correction (SVC) in a two sample t-test.

| **ROI** | **Contrast** | **ROI_max_** | **SVC_max_** | **T** | **p** |
| --- | --- | --- | --- | --- | --- |
| DMN.MPFC | ADHD>TDC | 1,55,-3 | -8 56 -4 | 3.2 | .036 |
| DMN.LP | ADHD>TDC | -39,-77,33 | -36 -84 30 | 3.0 | .049 |
|  | ADHD>TDC | 47,-67,29 | 54 -66 34 | 4.6* | .001 |
| DMN.PCC | ADHD>TDC | 1,-61,38 | -6 60 40 | 2.6 | .126 |
| FPN.LPFC | ADHD>TDC | -43,33,28 | -40,26,24 | 1.6 | .455 |
|  | ADHD<TDC | 41,38,30 | 36,38,38 | 1.6 | .450 |
| FPN.PPC | ADHD>TDC | -46,-58,49 | -48,-60,52 | 4.7* | .000 |
|  | ADHD>TDC | 52,-52,45 | 48,-56,54 | 3.6* | .011 |

**Note**. DMN: default mode network, DMN.MPFC: medial prefrontal cortex, DMN.LP: lateral parietal cortex, DMN.PCC: posterior cingulate cortex; FPN: fronto-parietal network, FPN.LPFC: lateral PFC, FPN.PPC: posterior parietal cortex.
