# Supplementary material for: Serotonergic modulation of normal and abnormal brain dynamics: The genetic influence of the TPH2 G-703T genotype and DNA methylation on wavelet variance in children and adolescents with and without ADHD
Source: PLoS One. 2023 Apr 27;18(4):e0282813. doi: 10.1371/journal.pone.0282813 (PMC10138254; doi:10.1371/journal.pone.0282813)
Supplement: S3 Table — ADHD: Attention Deficit/Hyperactivity Disorder, TDC: typically developing children, cogLoad: cognitive load, scale 3 = 0.0781–0.1562Hz. *: significant with pFDR < .004. (DOCX) [file pone.0282813.s007.docx]

**Table s3** effects of cognitive load on behavioral performance using repeated measures ANOVA (n_ADHD_=43. n_TDC_=56)

| **ROI** | **block** | **ADHD** | **TDC** | **F_group_** | **F_cogLoad_** | **F_groupXcogLoad_** |
| --- | --- | --- | --- | --- | --- | --- |
|  |  | **[M(SD)]** | **[M(SD)]** | **[F, p]** | **[F, p]** | **[F, p]** |
| **accuracy** | block1 | 73.7(15.3) | 75.0(18.1) | 1.1 | 0.1 | 0.2 |
|  | block3 | 72.1(21.3) | 80.4(18.7) |  |  |  |
|  | block4 | 76.4(19.0) | 79.1(19.4) |  |  |  |
|  | block5 | 73.3(17.0) | 75.0(18.5) |  |  |  |
| **errors** | block1 | 5.3(2.9) | 4.8(3.4) | 1.6 | 21.4* | 0.3 |
|  | block3 | 5.3(4.1) | 3.8(3.5) |  |  |  |
|  | block4 | 4.5(3.6) | 3.9(3.7) |  |  |  |
|  | block5 | 3.7(2.8) | 3.3(3.5) |  |  |  |
| **rt** | block1 | 472(116) | 423(95) | 6.1 | 1.2 | 0.1 |
|  | block3 | 405(92) | 379(55) |  |  |  |
|  | block4 | 453(102) | 422(80) |  |  |  |
|  | block5 | 469(97) | 421(63) |  |  |  |

**Note.** ADHD: Attention Deficit/Hyperactivity Disorder, TDC: typically developing children, cogLoad: cognitive load, scale 3=0.0781-0.1562Hz. *: significant with p_FDR_<.004.
