# Supplementary material for: Serotonergic modulation of normal and abnormal brain dynamics: The genetic influence of the TPH2 G-703T genotype and DNA methylation on wavelet variance in children and adolescents with and without ADHD
Source: PLoS One. 2023 Apr 27;18(4):e0282813. doi: 10.1371/journal.pone.0282813 (PMC10138254; doi:10.1371/journal.pone.0282813)
Supplement: S4 Table — DMN: default mode network, DMN.LP: lateral parietal cortex; FPN: fronto-parietal network, FPN.r.LPFC: right lateral PFC, FPN.r.PPC: right posterior parietal cortex; frequency bands: scale 3 = 0.08–0.16Hz, scale 5 = 0.02–0.041Hz. *: significant with pFDR<q* = .033; η2: partial eta squared with small effect size = 0.01; medium effect size = 0.06; large effect size = 0.14. (DOCX) [file pone.0282813.s008.docx]

**S4 Table.** Significant results of 2x2 MANCOVA models using *TPH2 genotype* and *group* as independent factors, *affective comorbidity* as covariate of interest*,* and ROI- and scale-specific wVar as dependent variables

|  | **F_TPH2_** | **F_group_** | **F_int_** | **F_comorb_** | **p-η^2^**_group_ |  |
| --- | --- | --- | --- | --- | --- | --- |
| **scale 3** | | | | | | |
| DMN.l.LP | 0.7, p=.409 | 6.5*, p=.013 | 0.1, p=.806 | 0.7, p=.407 | .097 |  |
| **scale 5** | | | | | | |
| FPN.r.LPFC | 0.4, p=.520 | 5.2*, p=.027 | 0.0, p=.974 | 0.7, p=.408 | .078 |  |
| FPN.r.PPC | 4.1, p=.048 | 5.8*, p=.019 | 0.7, p=.415 | 0.8, p=.370 | .088 |  |

**Note.** DMN: default mode network, DMN.LP: lateral parietal cortex; FPN: fronto-parietal network, FPN.r.LPFC: right lateral PFC, FPN.r.PPC: right posterior parietal cortex; frequency bands: scale 3=0.08-0.16Hz, scale 5=0.02-0.041Hz. *: significant with p_FDR_<q*=.033; η^2^: partial eta squared with small effect size=0.01; medium effect size=0.06; large effect size=0.14.
