# Supplementary material for: Serotonergic modulation of normal and abnormal brain dynamics: The genetic influence of the TPH2 G-703T genotype and DNA methylation on wavelet variance in children and adolescents with and without ADHD
Source: PLoS One. 2023 Apr 27;18(4):e0282813. doi: 10.1371/journal.pone.0282813 (PMC10138254; doi:10.1371/journal.pone.0282813)
Supplement: S5 Table — Note. DMN: default mode network, DMN.MPFC: medial prefrontal cortex; FPN: fronto-parietal network, FPN.r.LPFC/FPN.l.LPFC: right and left lateral PFC; frequency bands: scale 3 = 0.08–0.16Hz, scale 5 = 0.02–0.041Hz. p_eta2: partial eta squared with small effect size = 0.01; medium effect size = 0.06; large effect size = 0.14. (DOCX) [file pone.0282813.s009.docx]

**S5 Table. External validation results of univariate** ANOVA models using *group* as independent factor and ROI-and scale-specific **wVar from rs-fMRI timeseries only** as dependent variable (ADHD: N=124, no diagnosis given: N=126).

|  | **ADHD_rest_** | **TDC_rest_** | **F_group_** | **p_η^2^** |
| --- | --- | --- | --- | --- |
|  | [M(SD)] | [M(SD)] | [F, p] |  |
| **Scale 3** |  |  |  |  |
| DMN.MPFC | 0.09(.10) | 0.11(.16) | 2.6, p=.107 | .010 |
| FPN.l.LPFC | 0.06(.08) | 0.06(.07) | 0.1, p=.836 | .000 |
| FPN.r.LPFC | 0.06(.09) | 0.06(.06) | 0.1, p=.892 | .000 |
| **scale 5** |  |  |  |  |
| FPN.r.LPFC | 0.05(.07) | 0.06(.08) | 0.1, p=.763 | .000 |

**Note.** DMN: default mode network, DMN.MPFC: medial prefrontal cortex; FPN: fronto-parietal network, FPN.r.LPFC/FPN.l.LPFC: right and left lateral PFC; frequency bands: scale 3=0.08-0.16Hz, scale 5=0.02-0.041Hz. p_eta^2^: partial eta squared with small effect size=0.01; medium effect size=0.06; large effect size=0.14.
