# Supplementary material for: Serotonergic modulation of normal and abnormal brain dynamics: The genetic influence of the TPH2 G-703T genotype and DNA methylation on wavelet variance in children and adolescents with and without ADHD
Source: PLoS One. 2023 Apr 27;18(4):e0282813. doi: 10.1371/journal.pone.0282813 (PMC10138254; doi:10.1371/journal.pone.0282813)
Supplement: S1 Methods — (DOCX) [file pone.0282813.s010.docx]

**Electronic Supplementary Material**

**The experimental paradigm**

The task consisted of one baseline block outside the scanner (baseline: without the option of earning a reward) and five experimental runs within the scanner including a second baseline block (block 2). In the task, subjects were instructed to indicate the position of a briefly presented visual target after a waiting period to earn a monetary reward. An experimental trial started with the presentation of a visual ‘cue’ i.e., the presentation of the 4 boxes/choices, representing the start of the trial and initiating the waiting period. After a certain cue-target interval the ‘target’ appeared in terms of a green circle in one of the four choices. After the subject’s response, the reward was calculated: in the baseline block outside the scanner the average reaction time window of each subject was determined (M±1SD). If the subjects responded correct and within their time windows, they received 10Eurocent, if they responded correct and faster than their time windows, they received 1Eur and if they were slower, they lost 1Eur. Incorrect responses were neither punished nor rewarded. According to the calculated reward, feedback was presented showing the amount of recently earned/lost money in combination with the overall amount of earned money (**figure s1, left side**). The subjects were instructed to press the corresponding button as fast and as correct as possible.

Across experimental runs, task difficulty was increased as follows:

**block 3:** reduction of the target’s presentation duration from 64ms to 32ms

**block 4:** short target presentation + variation of the cue-target interval between 2000ms and 6500ms, **block 5:** short target presentation + variation of the cue-target interval + inclusion of distractor targets in terms of targets with blue and yellow circles preceding the actual target (**figure s1, right side**).

A scanning session started with two training sessions of 10 trials each and a baseline run of 20 trials outside the scanner. In the MR-scanner 5 blocks of 20 trials were presented, separated by breaks of 40sec each. The baseline run outside the scanner lasted 2.5min, the part within the scanner took 14.5min.
